# Multi-Parameter Characteristics of Electric Arc Furnace Melting

**DOI:** 10.3390/ma15041601

**Published:** 2022-02-21

**Authors:** Michał Moskal, Piotr Migas, Mirosław Karbowniczek

**Affiliations:** 1 COGNOR SA Branch HSJ in Stalowa Wola, Kwiatkowskiego 1, 37-450 Stalowa Wola, Poland; 2 Department of Ferrous Metallurgy, Faculty of Metals Engineering and Industrial Computer Science, AGH-University of Science and Technology, 30 A. Mickiewicza A., 30-059 Krakow, Poland; pmigas@agh.edu.pl (P.M.); mkarbow@agh.edu.pl (M.K.)

**Keywords:** electric arc furnace, energy efficiency modelling, process optimization, mass and energy balances

## Abstract

The article presents the results of analyses of numerical modelling of selected factors in electric arc furnace melts. The aim of the study was to optimise the melting process in an electric arc furnace using statistical-thermodynamic modelling based on, among other things, multiple linear regression (MLR). The article presents tools and methods which make it possible to identify the most significant indicators of the process carried out on the analysed unit from the point of view of improvement. The article presents the characteristics of the process and creation of the MLR model and, by applying its numerical analyses and results of calculations and simulations for selected variables and indicator, identifying the operation of a selected furnace. Developed model to demand of electric energy identification was used for calculations of energy balances, the distribution of the energy used in the furnace was presented.

## 1. Introduction

Observed climatic changes in the environment are causing recycling technologies for steel production to increase their share in the total balance of crude steel production. This is particularly noticeable in the European Union. Therefore, the electric arc furnace is becoming one of the leading processing units for steel scrap used in crude steel production [1]. Electricity consumption in this process still accounts for about 50–80% of the total energy consumption during melting. Technological progress and the development of information technology create opportunities to reduce the unit demand for electrical or chemical energy.

Mathematical descriptions and characterisations of the arc furnace smelting process are complicated due to its nature resulting from, among others, high temperature and chemical composition gradients, turbulent flows, multiphase and heterogeneity of the system, significant mass flow of substrates, high speed of phase transformations and non-linearity of phenomena. Therefore, the development of modelling of selected stages or the entire arc furnace smelting process remains justified [2].

The models used to describe the melting technology can be physical models—reproducing the processes on a smaller scale (e.g., laboratory and/or using other media than liquid metal and slag with the use of criterion numbers). An example of the application of a high-temperature model can be the studies on the performance of burners in an electric arc furnace carried out by Yonmo Sung [3]. They presented modification of oxy–gas burners, which was validated on a test bench.

Studies of scrap melting phenomena were also carried out based on water models [4] (using ice, three heating rods, and steam nozzles, which reflected scrap, electric arc, and oxy–gas burners, respectively).

Numerical methods can also be used to model the process. Mombeni [5] used a CFD model calculated in the commercial software ANSYS FLUENT to address the crack formation of water-cooled panels installed in the furnace vault. CFD modelling can also be used to determine the temperature distribution in a metal bath in the context of burner placement and media flow rates [6].

However, the mathematical models are the most universal method, which take into account physicochemical processes, thermodynamic and theoretical equations, mass and energy balances, or only technological data from the process [7]. They can be divided into two categories: linear and non-linear.

Linear models are created as a linear combination of predictor variables [8] based on average statistical input and output data from the process, or assumed theoretical values. They describe the relationship between individual parameters and their importance for the outcome of the calculation. Linear models are static and refer to only two points in time; they are the result of input data and, as mentioned above, they can only be used with reference to average values of a given process [9].

The most popular is the Kohle model, which describes the influence of process parameters on electricity demand.

Another model based on a multivariate linear regression equation is the one developed by Haupt [10]. He determined the influence of scrap quality on the melting process in an electric arc furnace.

However, non-linear models have the ability to describe the process more accurately. R. Morales created one of the first models, which included heat and mass transfer calculations and chemical reaction kinetics in terms of use the direct reduced iron [11]. The author created a dynamic model, which took into account the relationships between individual phenomena in terms of their physical course and not just the result, which was a statistical outcome of many variables. Thus, it makes it possible to analyse single melts and to follow the course of change of the result in time.

Attempts have been made to simplify the models and organise their construction. Clear boundaries of individual zones in the furnace were set. In terms of both: furnace construction, melting stages, and division into individual phases of materials/substances present in the furnace space. Bekker [12] specified two groups of solids and liquids in the constructed model. On the other hand, Logar [13,14] created a system of dependent modules representing particular groups of phenomena (including solid scrap zone, solid slag zone, liquid slag zone, liquid metal zone, and wall and roof zone). Models using neural networks are becoming increasingly popular. The first versions were developed in the 1990s. Ledoux and Bonnard, in their work, built a model based on a multi-layer perceptron. The model described the dynamics of an electric arc during the melting process.

Due to the process complexity, it is necessary to select models in such way that sufficient representation of the processes is maintained with the best possible ability to interpret and understand the simulation results [15]. The balance between these two characteristics depends largely on the type of models and the modelling methods [16].

## 2. Materials and Methods

### 2.1. Theoretical Mathematical Models

Mathematical models are the most commonly used in EAF simulation. They can be both linear and non-linear in nature. This classification results from taking into account the above-described variability of process efficiency as well as the variability of the parameters themselves during the melt [17]. Taking into account the kinetics of the reaction or the variation in the efficiency of media use or their control transforms the model into a non-linear form. Non-linearity is understood as effects ranging from media consumption dependent on melting programmes to the dependence of heat losses on the number and height of scrap heaps and thus arc exposure. When creating mathematical models, theoretical data or available technological and statistical data are used—which, depends on their availability and quality.

### 2.2. Linear Modelling of Steel Melting in Electric Arc Furnace

Regression linear models are created as a linear combination of predictor variables based on statistical mean input and output data from a process, or assumed theoretical values. This means that they refer to (only) two points in time, they are the result of input data and, e.g., output data, and as mentioned above, and they can only be used with respect to the average values of a given process. Static models often do not take into account some process disturbances and micro-scale phenomena (of low weights) and, therefore, their use for the analysis of single melts becomes difficult or, even impossible, due to the high variability of the real process conditions [18]. 

The most widely used statistical model in the literature is the Köhle model [18,19]. Köhle’s Equation (1) describing the demand for electricity:

(1) $$\frac{W_{r}}{\mathrm{kWh}/t}=300+900\left[\frac{G_{E}}{G_{A}}-1\right]+1600\frac{G_{Z}}{G_{A}}+0.7\left[\frac{T_{A}}{{}^{\circ}C}-1600\right]+0.85\frac{t_{S}+t_{N}}{\min}-8\frac{M_{G}}{m^{3}/t}-4.3\frac{M_{L}}{m^{3}/t}$$

where W_r_ is the electric energy demand, kWh/Mg; G_E_ is the weight of ferrous materials, kg; G_A_ is the tap weight (mass of the bath), kg; G_Z_ is the weight of slag components, kg; T_A_ is the temperature before taping, °C; t_S_ is the power on time, min; t_N_ is the power off time, min; M_G_ is the natural gas consumption, Nm^3^; and M_L_ is the oxygen consumption, Nm^3^.

This model characterises the electric energy demand, which in this case (of the units analysed by Köhle) is between 380 and 600 kWh/Mg with an accuracy equal to the standard deviation of 5 kWh/Mg. 

The disadvantage of this solution is the low sensitivity of the model to changes in process conditions (occurrence of disturbances, change of the melting programme for the arc control system, or burners) which translates into a high error for individual melts. However, it is also an advantage in terms of long-term prediction. The Köhle model, due to its hybrid empirical–theoretical character, remains easy to interpret while maintaining sufficient accuracy.

In industrial practice, problems are encountered due to, e.g., insufficient process metering and difficult definition of boundary conditions. The amount of electrical energy supplied is easy to determine, whereas the efficiency of heat transfer through the arc depends on a large number of factors, e.g., the melting stage, the phenomena associated with arc burning (arc length, arc stability), or the plasma temperature associated with the gas atmosphere of the furnace, as well as the parameters characterising the steel scrap. Identifying the efficiency of the use of chemical energy—dependent on the reactions taking place in the reactor, which is the volume of the furnace bowl—is also a challenge. Its determination requires the identification of the reactions taking place and the distribution of the heat obtained between the phases, as well as the dynamics of the process [11,12,13,20].

When constructing models, it is necessary to perform mass and energy balances. Carrying out correctly defined balance calculations (of mass and energy) is essential from the point of view of the overall characteristics of the energy efficiency of the process and the determination of process boundary conditions [11].

In the course of the analyses, a database of indicators and technological parameters was created, which included 1953 records from one specific EAF. Due to a wide range of steel grades, the database was based on the most frequently manufactured group of steel grades and data was selected by rejecting melts:

- from furnace start-up periods;
- with furnace operation disturbances;
- switching from or to grades with large differences in chemical composition, when there was a justified suspicion of wet pouring;
- with process parameter values that are outliers.

The parameters included in the database are batch, material, and energy parameters.

### 2.3. Mass Balances

The mass balance is based on the assumption that the sum of the masses of the inputs m_in_ equals the sum of the outputs m_out_.

(2) $$m_{\mathrm{scr}}+m_{\mathrm{sf}}+m_{c}+m_{\mathrm{fa}}+m_{\mathrm{ox}}+m_{{\mathrm{CH}}_{4}}+m_{\mathrm{la}}=m_{\mathrm{hm}}+m_{\mathrm{sl}}+m_{f}+m_{\mathrm{wg}}$$

where m_scr_ is the mass of the scrap, kg; m_sf_ is the mass of the slag formers, kg; m_ox_ is the mass of the oxygen, kg; m_CH4_ is the mass of the natural gas, kg; m_c_ is the mass of the carburizer, kg; m_fa_ is the mass of the foaming agent, kg; m_la_ is the mass of the leaked air, kg*;* m_hm_ is the mass of the hot metal, kg; m_sl_ is the mass of the slag, kg; m_wg_ is the mass of the waste gases, kg; and m_f_ is the mass of the fumes, kg.

For the purposes of consideration, the assumption has been made that the mass of the hot heel does not change between heats. In reality, the mass of hot heel changes and is difficult to estimate due to the relatively small volume it occupies and the constant irregular changes in the scale changing the volume of the furnace hearth.

#### 2.3.1. Slag Weight Calculations

Slag mass was calculated on the basis of the chemical composition of the scraps used, assuming that the elements contained in them: Si, Mn, Cr, C, P, and Al are oxidised to the average of the level characterising the composition of the metal bath, and the amount of oxidised Fe constitutes 5.5% of the charge mass. These elements and the oxygen needed to burn them together with the addition of CaO and MgO make up the slag mass. The slag mass also includes ash from carbon carriers.

Chemical compositions of the slags were identified via energy dispersive X-ray fluorescence (XRF Oxford X-MET 3000TX +). In order to identify the chemical composition of the steel, a spectrophotometric method of analysis was used on a FOUNDRY-MASTER spark spectrometer. 

The correctness of assumptions and calculations was checked by calculating the slag mass on the basis of CaO introduced into the process, which, as a component not dissolving in the metal bath, theoretically remains entirely in the slag.

(3) $$m_{\mathrm{sl}}=\frac{\left(m_{\mathrm{CaO}\;\mathrm{met}.}\cdot 0.958+m_{\mathrm{CaO}\;\mathrm{inj}.}\cdot 0.864\right)\cdot 100\%}{{\%\mathrm{CaO}}_{\mathrm{slag}}}$$

where m_CaO met._ is the mass of the metallurgical calcium, kg; m_CaO inj_. is the mass of the injected pulverized calcium, kg; and %CaO_slag_ is the percentage of mass weight of CaO in slag.

A calculation was then made of the mass of each element that was oxidised during bath melting and slag formation, thus reducing the metal yield.

(4) $$m_{\mathrm{ae}}=\frac{M_{A_{e}}\cdot \%\mathrm{tl}\;\cdot {\;m}_{\mathrm{sl}}}{M_{A_{o}}}.\;$$

where m_ae_ is the mass of the alloying element melted in slag, kg; %tl is the percentage of mass weight of given element in slag; $M_{A_{e}}$ is the atomic mass of element, u; and $M_{A_{o}}$ is the atomic mass of oxide of given element, u.

#### 2.3.2. Calculation of Waste Gas Masses

In order to calculate the mass of waste gases taking into account the course of the reaction of combustion of coal and methane, Reactions (5)–(7) were written:

(5) $$C+\frac{1}{2}O_{2}\to \mathrm{CO}$$

(6) $$C+O_{2}\to {\mathrm{CO}}_{2}$$

(7) $${\mathrm{CH}}_{4}+2O_{2}\to {\mathrm{CO}}_{2}+2H_{2}O$$

The mass of carbon monoxide and carbon dioxide for each reaction for one kilogram of product burnt was calculated. The values obtained were, respectively, 2.33 kg, 3.67 kg, and 2.75 kg (from Reactions (5)–(7)). Due to the lack of measurement of the flue gas composition, it was assumed that all the substrates burned completely, and left entirely with the flue gases. Therefore, in the mass of waste gasses m_wg_, the components according to the Equation (8) are included:

(8) $$m_{\mathrm{wg}}=m_{N_{2}}+m_{{\mathrm{CO}}_{2}}+m_{H_{2}O}$$

#### 2.3.3. Calculation of Metal Bath Weight

The theoretical weight of the metal bath was calculated as the difference between the weight of the scrap, the alloying elements contained in the bath (including iron), and the estimated amounts of fumes.

(9) $$m_{\mathrm{hm}}=m_{\mathrm{scr}}-{\Sigma m}_{\mathrm{ae}}-m_{f}$$

where m_hm_ is the mass of hot metal, kg; m_scr_ is the mass of the scrap, kg; m_ae_ is the sum of masses of alloying elements that went into slag, kg; and m_f_ is the mass of the fumes, kg.

### 2.4. Energy Balances

The energy balance assumes that the sum of the energy input E_in_ into the process is equal to the sum of the energy output from the process in the form of product heat Q_out_ and thermal losses Q_loss_. The energy input to the system consists of electrical energy and chemical energy. In the other direction, the energy is transferred out of the process in the form of metal baths, liquid slag, waste gases, dusts, and heat losses.

(10) $$E_{\mathrm{el}.}+E_{\mathrm{chem}.}=Q_{\mathrm{hm}}+Q_{\mathrm{sl}}+Q_{\mathrm{wg}}+Q_{f}$$

where E_el._ is the electrical energy, MJ; E_chem._ is the chemical energy, MJ; Q_hm_ is the heat of hot metal, MJ; Q_sl_ is the heat of the slag, MJ; Q_wg_ is the heat of waste gases, MJ; and Q_f_ is the heat of the fumes, MJ.

The chemical energy was calculated on the basis of enthalpies of exothermic reactions—oxidation of bath components and combustion of methane. The Perry Nist Janaf method was used to calculate the enthalpies of the reactions. 

#### 2.4.1. Calculation of the Physical Heat of Hot Metal

Calculation of the physical heat of the hot metal is based upon the values of the specific heat of the hot metal, its mass, and the temperature rise. It also takes into account the phase transition by using the values of latent heat, liquid temperature, and differentiation of specific heat into that occurring in the liquid and solid state. 

For the calculation of the physical heat of the metal bath, the Formula (11) is used:

(11) $$Q_{\mathrm{hm}}=m_{\mathrm{hm}}\cdot \left(c_{\mathrm{ss}}\cdot \left(T_{m}-T_{a}\right)+Q_{\mathrm{lst}}+c_{\mathrm{ls}}\left(T-T_{m}\right)\right)/1000$$

where, Q_hm_ is the physical heat of the metal bath at temperature T, MJ; m_hm_ is the mass of hot metal, kg; c_ss_ is the average specific heat of steel in the solid state, kJ/kg K; c_ls_ is the average specific heat of steel in the liquid state, kJ/kg·K; Q_lst_ is the latent heat of fusion of steel, kJ/kg; T is the steel tap temperature, K; T_m_ is the steel melting temperature, and K; T_a_ is the ambient temperature, K.

The specific heat of the liquid state was taken as 0.836 MJ/Mg*·*K and that of the solid state as 0.698 MJ/Mg*·*K. The latent heat of fusion for the steel was taken as 271.7 kJ/kg.

The melting point of steel with a hot metal composition was calculated according to the Formula (12):

(12) $$T_{m}=T_{\mathrm{Fe}}-\overset{n}{\underset{i=1}{\sum }}p_{i}k_{i}$$

where T_m_ is the melting point, K; T_Fe_ is the melting point of pure iron (1812 K); p_i_ is the percentage content of the element in the metal bath, %; and k_i_ is the temperature reduction factor (according to the Table 1) [21].

#### 2.4.2. Calculation of the Physical Heat of Slag

The calculation of the physical heat of slag was based on the values of the slag’s specific heat, mass, temperature rise, and latent heat. The following Formula (15) was used to calculate the physical heat of slag:

(13) $$Q_{\mathrm{sl}}=\frac{m_{\mathrm{sl}}\left(c_{\mathrm{sl}}\left(T-T_{a}\right)+Q_{\mathrm{lsl}}\right)}{1000}$$

where Q_sl_ is the physical heat of slag at temperature T, MJ; m_sl_ is the slag mass, kg; c_sl_ is the specific heat of slag at temperature T*,* kJ/kg·K; Q_lsl_ is the latent heat of melting of slag, kJ/kg; T is the slag tap temperature, K; and T_a_ is the ambient temperature, K.

The specific heat of slag was calculated from the Formula (14):

(14) $$c_{\mathrm{sl}}=0.736+2.93\cdot 10^{-4}T$$

The assumed latent heat of fusion is 209 kJ/kg.

#### 2.4.3. Calculation of the Physical Heat of Waste Gases

The calculation of the physical heat of the waste gases is based on the specific heat values of the gases of their mass and temperature rise. The formula was used to calculate the physical heat of the waste gases:

(15) $$Q_{\mathrm{wg}}=m_{\mathrm{wg}}c_{\mathrm{wg}}\left(T-T_{a}\right)/1000$$

where Q_wg_ is the physical heat of the waste gases at temperature T, MJ; m_wg_ is the mass of the waste gases, kg; c_wg_ is the specific heat of the waste gases at temperature T, kJ/kg·K; T is the temperature of the gases, K; and T_a_ is the ambient temperature, K.

The average specific heat of the waste gases is 1.33 kJ/Nm^3^, whereas 1450 °C was taken as the value of gas temperature.

## 3. Results

In the field of research, considering modelling (MLR) as a first step, an attempt was made to apply and subsequently modify the Köhle equation [22]. Based on the analyses carried out on a furnace operating under the industrial conditions, one should state the lack of universality for the form of the model proposed by Köhle. The obtained values differ from the measured ones by an average of 50% for the 1992 version and by an average of 10% for the 2002 version of the equation [23]. As part of the work performed, the model was modified, and one of the changes made was the replacement of the M_L_ coefficient relating to oxygen consumption by an equation taking into account the heat gain from the oxidation of individual elements. This is due to the previously mentioned large number of scrap classes, as well as the large share of the iron oxidation reaction as a source of chemical energy. Therefore, the replacement of the coefficient that represents the average heat gain by a separate function is justified. This function is written in the form:

(16) $$E_{M}=\sum_{M}\left(M_{\mathrm{sl}}\cdot \frac{M_{\mathrm{AM}}\cdot {\mathrm{avg}\%}_{\mathrm{MO}.\mathrm{sl}}\cdot 1000\frac{\mathrm{kg}}{\mathrm{Mg}}}{100\%\cdot M_{\mathrm{AMO}}}\cdot {\Delta H}_{M}\right)$$

where M_sl_ is the mass of slag, Mg; M_AM_ is the atomic weight of considered metal, u; avg%_MO.sl_ is the average considered metal oxide content of slag, %; M_AMO_ is the atomic weight of considered metal oxide, u; and ΔH_M_ is the the enthalpy of the oxidation reaction of a considered element, converted to kWh/kg; (where M = Cr, Mn, Fe, Al, Si).

**Figure 1:**
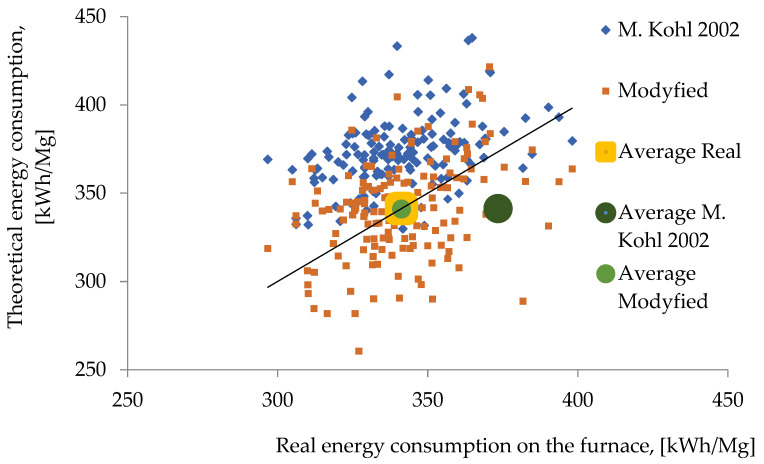
Comparison of Köhle model results with real electricity consumption [23].

Figure 1 shows the comparison of the real values of electricity consumption with the model values obtained based on two versions of the Köhle model: the 2002 version and its modification. It can be noted that for the modified version a much higher model fit was obtained (average difference 1%, maximum 20%).

In the realities of the furnace characterised, the model did not adequately provide quantitative answers as to what steps and extent of change should be taken to optimise the process. 

Therefore, mass and energy balances were calculated and a linear multiple regression (MLR) model was undertaken.

For the calculation of mass and energy balances, it was necessary to identify the chemical composition of the liquid products (slag and steel). For this purpose, slag and metal samples were taken. A study of the chemical composition of slags was carried out for 200 melts in the same group of steel grades. The obtained average chemical compositions of slag and steel are presented in Table 2 and Table 3.

In the case of the unit under consideration, the insufficient metering, the high diversification of the steel grades, and the high variability of the charge (based on more than 40 scrap grades) led to some simplifications. For example, assuming a constant hot heel level; ignoring the change in furnace geometry resulting from erosion of the lining and, thus, assuming that the oxygen stream always reaches the bath and is fully consumed; and assuming that the chemical composition of the scrap corresponds to the average of the ranges described in PN 85/H 15000.

The results of the mass and energy balances obtained, and their analysis, clearly indicate the areas which should be investigated in order to improve the scrap melting process, e.g., appropriate use of natural gas, problems with furnace leak-tightness, and use of oxygen. However, the predominance of chemical energy in the melting process suggests that its optimisation will have a significant impact on the overall energy balance of the melt.

The parameters used to create the MLR were time, which is related to the amount of heat loss in the process; oxygen and gas consumption, as sources of chemical energy; the amount of slag-forming materials used, whose melting requires a significant amount of energy; and the scrap yield, which gives information on the amount of iron oxidised.

Calculations and multiple regression analyses were carried out to obtain the equation identifying the demand for electric energy. Table 4 presents the results for the selected backward MLR analysis, which gave the selected statistical model—the form of Equation (17). Statistical calculations were performed for the total sample size, which was N = 1959; the significance level of α = 0.05 was assumed. Statistical correlation between the considered independent variables was checked; the relationships between the variables present in the equation were statistically insignificant. 

Statistical results were obtained: the regression coefficient at the level of R = 0.497; comparing for R_table_ = 0.1946 (for N > 1000), one can state the significance of the calculated linear correlation. Fisher–Snedecor’s F-value, at the α = 0.05 level, F(5.1953) = 128; for Ho’s hypothesis, comparing it with the table critical value of F_table_ = 2.108, H_o_’s hypothesis of statistical significance R^2^ of the equation—can be accepted at the significance level of α = 0.05, which implies the possibility of rejecting the alternative hypothesis. It can also be seen that for all selected variables, the probability *p* is less than the significance level of 5% (*p* < α), and the independent variables present in the equation are individually statistically significant.

Following the above, a linear regression model was created (3), resulting in:

(17) $$E_{\mathrm{el}}=169.94+0.26\cdot t_{\mathrm{TTT}}+3.27\cdot \frac{m_{\mathrm{ox}}}{m_{\mathrm{scr}}}-1.98\cdot \frac{m_{{\mathrm{CH}}_{4}}}{m_{\mathrm{scr}}}+0.11\cdot \frac{m_{\mathrm{sf}}}{m_{\mathrm{scr}}}+48.79\cdot \frac{m_{\mathrm{hm}ł}}{m_{\mathrm{scr}}}$$

where t_ttt_ is the tap to tap time, min.; m_scr_ is the mass of scrap in charge, kg; m_ox_ is the mass of oxygen, kg; m_CH4_ is the mass of natural gas, kg; m_sf_ is the mass of slag formers, kg; and m_hm_ is the mass of hot metal, kg 

**Figure 2:**
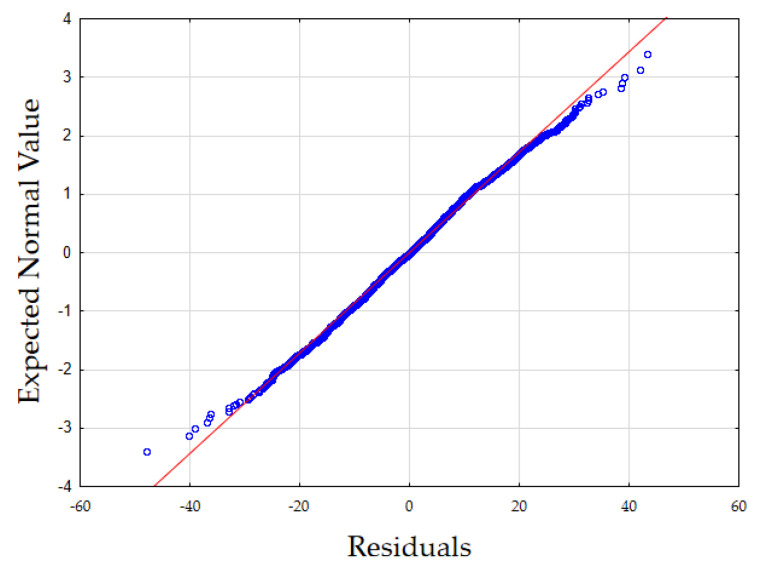
Normal distribution of residuals.

In Figure 2, the normality analysis of the distribution of the residuals was shown. It seems, Shapiro–Wilk test: SW–W = 0.9971, *p* = 0.0009. Variance constancy: fulfilled, the uniformity of the scatter of the residuals is constant across the width of the interval.

Simulations using the created MLR model confirmed the conclusions of the analysis of the balances performed, i.e., the predominance of chemical energy and the inverse proportionality of methane consumption to electrical energy consumption. 

Taking into account the analysis results, which confirmed the conclusions of the mass and energy balance, the description of the process efficiency (defined as the ratio of the supplied energy to the amount of physical heat of the metal bath) as a function of the share of chemical energy was undertaken. 

**Figure 3:**
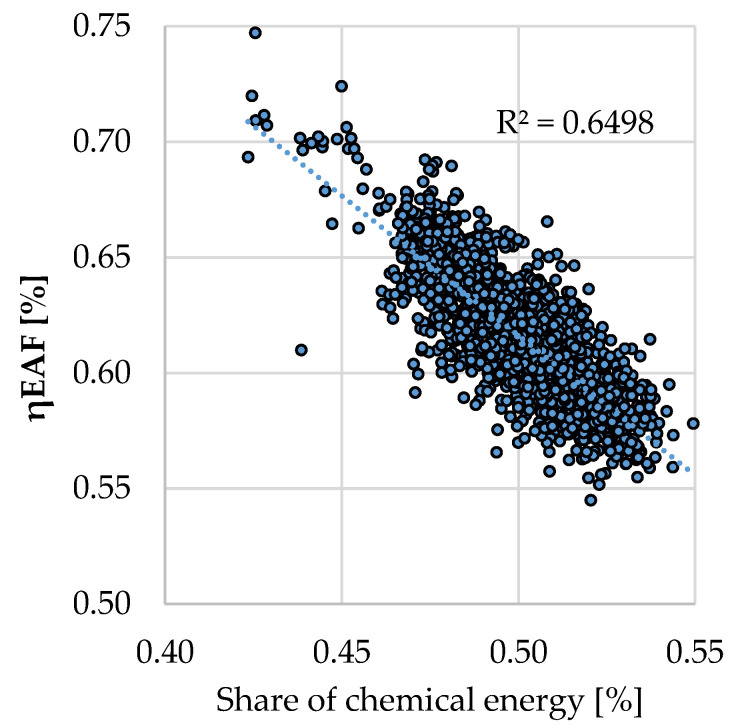
Dependence of process efficiency on the share of chemical energy.

In Figure 3, the analysis of the dependence of process efficiency on the share of chemical energy was shown. A decrease in process efficiency (η_EAF_) is visible with an increase in the share of chemical energy in the process. This trend in melt efficiency prompted an analysis of the efficiency of chemical energy use in the process under study.

As an example, the amount and timing of CH_4_ addition was analysed as a function of the efficiency of the heat generated by its combustion. 

**Figure 4:**
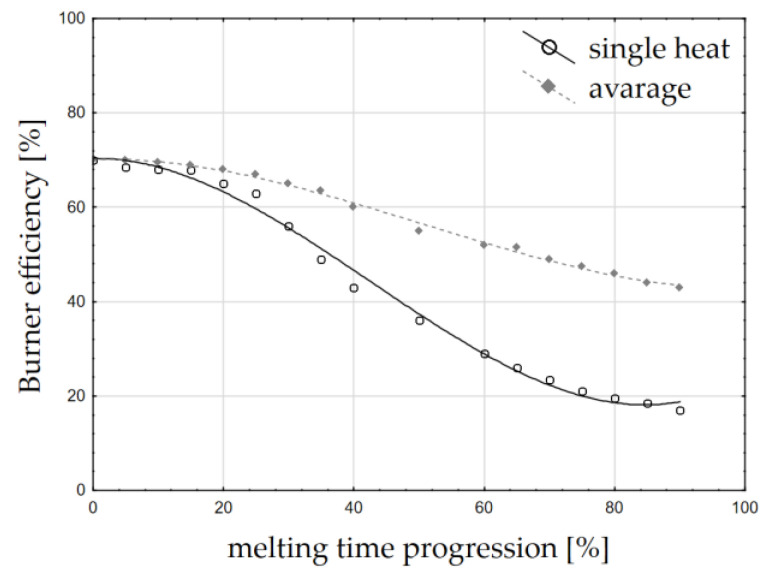
Burner efficiency as a function of melting time progression [%] (own study based on data from [21]).

It was assumed that the efficiency will change with the progress of scrap melting. This is due to phenomena such as a change in the scrap surface with the progress of melting, but most importantly due to a decrease in the temperature difference between the flame and the charge, which heats up during the process. The change in burner efficiency was re-calculated on the basis of the approach presented in [21,24,25] and is presented in Figure 4. 

The energy efficiency of the burners was considered first. This varies with the progress of melting. This efficiency is, on average, between 70 and 40%, and can drop as low as 20%. To identify the energy transfer efficiency, the Gottardi methodology was used [25], which made the energy efficiency of the burner dependent on, among other things, the temperature difference between the flame and the charge, which decreases with the progress of melting and, additionally, with a decrease in the scrap surface area. This dependence is described by the function (18):

(18) $$\eta_{{\mathrm{CH}}_{4}}=70.272-8\cdot 10^{-3}\cdot p_{t}^{2}+5\cdot 10^{-5}\cdot p_{t}^{3}$$

where $\eta_{{\mathrm{CH}}_{4}}$ is the energy efficiency of the burner [%]; and p_t_ is the melting progression [%].

Based on the mathematical description of the change in burner efficiency as a function of melting progress, three patterns of energy gain as a function of the average gas flow possible under the conditions of the unit analysed were simulated. Boundary conditions were established: minimum gas flow in burner mode, 90 Nm^3^/h; the currently used average gas flow rate, 110 Nm^3^/h; and near-maximum flow rate, 200 Nm^3^/h. The maximum burner operation time—resulting from the melting stage characterised by the presence of scrap in the “cold regions” of the walls—is 30 min in total for all the baskets. 

**Figure 5:**
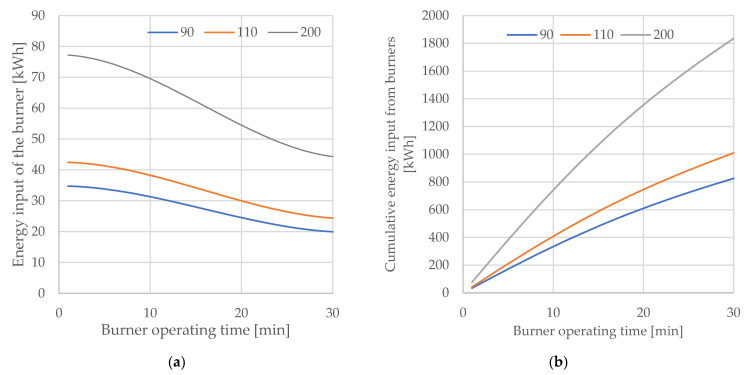
(**a**) Variation of burner energy input as a function of burner operating time (**b**) cumulative energy as a function of burner operating time

The change in energy contributed by the burners over time (Figure 4), the cumulative energy per 30 min (Figure 5a), and the cumulative energy as a function of the maximum possible amount of gas supplied (Figure 5b) were considered. 

From the simulations obtained, it can be concluded that it is beneficial to intensify the gas flow in the initial stage of the melt, as this allows the burner to use its moment of greatest efficiency. The calculated maximum achievable gas consumption is, respectively, 135, 165, and 300 Nm^3^, and the energy contributed is 826 kWh, 1009 kWh, and 1835 kWh.

Finally, the equation was created to simulate production costs (in the sense of direct manufacturing costs), CO_2_ emissions, and process efficiency (Mg of hot metal/h)—it is assumed that such a construction of the model will allow to control the technology in a manner depending also on economic objectives.

The attempt to consolidate the results in terms of obtaining the unit melt cost function contributed to the creation of Equation (19).

(19) $$P_{c}=E_{\mathrm{el}}\cdot X_{1}+m_{{\mathrm{CH}}_{4}}\cdot X_{2}+\left(m_{\mathrm{scr}}-m_{\mathrm{hm}}\right)\cdot X_{4}+m_{\mathrm{ox}}\cdot X_{3}+\left(m_{C}+m_{{\mathrm{CH}}_{4}}\right)\cdot \frac{44}{16}\cdot X_{5}+m_{\mathrm{sf}}\cdot X_{6}$$

where P_c_ is the production cost; X_1_ is the cost per unit of electrical energy, PLN/kWh; X_2_ is the cost per unit of natural gas, PLN/Nm^3^; X_3_ is the cos per unit of technical oxygen, PLN/Nm^3^; X_4_ is the average unit cost of scrap purchase, PLN/Mg; X_5_ is the cost per emission of CO_2_ unit PLN/Mg; and X_6_ is the cost per unit of slag formers, PLN/kg.

**Figure 6:**
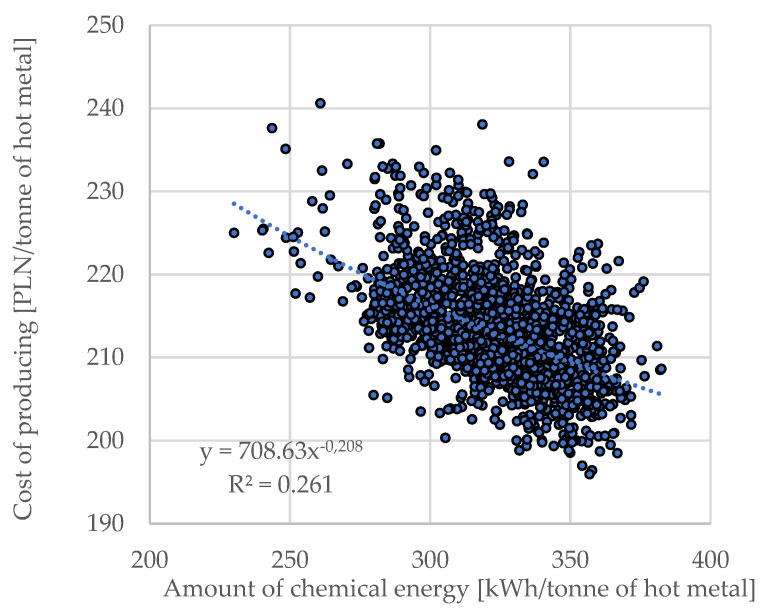
The amount of chemical energy in relation to the direct cost of producing a tonne of metal bath.

Equation (5) was used to calculate production costs (in terms of direct production costs of liquid steel), CO_2_ emissions, and process efficiency (Mg of hot metal/h)—assuming that such a model construction would allow to control the technology in a manner depending also on economic objectives. After calculating the production costs for the smelters under consideration (forming the database), a correlation analysis was carried out between the production cost (less the price of scrap metal forming the metal bath) and the amount of chemical energy input; the results are presented in Figure 6.

The correlation coefficient is R = 0.513 and the *t*-Student’s *t*-value reaches *t* = 41.51 with t_tabular_ = 1.960. From the existence of this relationship (Figure 6) it should be concluded that it is advantageous to increase the share of chemical energy in the process, but taking into account the efficiency of its transfer by the oxygen treating bath stage.

## 4. Discussion

Modelling of the melting process in an electric arc furnace is possible under industrial conditions, even with incomplete metering. However, the individual characteristics of the unit under consideration have to be taken into account as existing models do not show universality. The use of models developed for other units requires their modification and taking into account the characteristics resulting from the proportion and share of chemical and electric energy as well as the input and technological conditions of the furnace. 

Increasing the chemical energy input into the melt lowers the required total amount of electrical energy—methane consumption is inversely proportional to electrical energy consumption. However, due to the technological effect of the fact that oxygen is fed most intensively at the time of charge reheating—throughout this period—its correlation with electrical energy is positive.

Based on model calculations for the analysed furnace unit using a modified Köhle model from 2002, the model and actual data converged at the level of R = 0.36. The calculated electricity demand differed from the actual demand at the maximum level of 20%. However, it did not give sufficient guidance for process control.

Thanks to the mass and energy balance calculations performed, results were obtained from which it can be concluded that there is a predominance of chemical energy (52%) in the process compared to electrical energy. 

It can be concluded that it is advantageous to intensify the gas flow at the initial stage of melting, as this makes it possible to use the moment of maximum efficiency of the burners (presented on Figure 5a,b). These conclusions require a complementary analysis; the real conditions of the process should be taken into account, which limit the maximum flow on the oxy–gas burners in the initial stages of the process (risk of burner damage by flame reflection from the scrap metal near the walls) and the fact that a higher energy input will lead to a faster melting of the deposited scrap metal

The statistical calculations carried out and the analyses of the statistical indices obtained made it possible to select the multiple linear regression model Equation (17)—as a method giving the possibility of an easy interpretation with an appropriate representation of the process in these conditions. The regression coefficient for the calculated model is R = 0.497, the difference between the calculated value and the real one is, at maximum, 17% (with mean 0). The obtained model was extended by the analysis of natural gas efficiency and consolidated with the cost calculation equation.

## Figures and Tables

**Table 1:** Temperature reduction factor for individual elements [21].

Element	C	Mg	Si	P	S	Cu	Ni	V	Mo	Cr
k*_i_*	65	5	8	30	25	5	4	2	2	1.5

**Table 2:** Average chemical composition of EAF slags before tap.

Oxide	CaO	MgO	MnO	Cr_2_O_3_	SiO_2_	P_2_O_5_	Al_2_O_3_	TiO_2_	FeO
% of weight	29.54	5.71	9.09	7.52	9.55	0.41	1.19	0.42	36.57

**Table 3:** Average chemical composition of liquid bath before tap.

Element	C	Mn	Cr	Ni	Si	P	S
% of mass	0.071	0.083	0.107	0.109	0.005	0.008	0.107

**Table 4:** Statistical parameters of calculated MLR model.

Variable	b*	Std. Err. of b*	B	Std. Err. of b	t(1953)	*p*-Value
Intercept			169.942	8.689	19.558	0.000
Tap-to-tap time	0.248	0.020	0.263	0.021	12.324	0.000
Oxygen to charge	0.403	0.020	3271	0.166	19.670	0.000
Natural gas to charge	−0.055	0.020	−2633	0.952	−2767	0.006
Slag formers to charge	0.064	0.020	0.113	0.036	3166	0.002
Scrap yield	0.123	0.021	48.787	8.377	5.82	0.000

b* is a structural parameters of the regression function; B is an estimators of obtained regression equation; t is a t-Student test probability distribution; and *p*-value is a probability level of variable.

## Data Availability

Not applicable.
